# Effect of Feedback during Virtual Training of Grip Force Control with a Myoelectric Prosthesis

**DOI:** 10.1371/journal.pone.0098301

**Published:** 2014-05-27

**Authors:** Hanneke Bouwsema, Corry K. van der Sluis, Raoul M. Bongers

**Affiliations:** 1 University of Groningen, University Medical Center Groningen, Center for Human Movement Sciences, Groningen, The Netherlands; 2 University Medical Center Groningen, Center for Rehabilitation, Groningen, The Netherlands; SUNY Downstate MC, United States of America

## Abstract

The aim of this study was to determine whether virtual training improves grip force control in prosthesis use, and to examine which type of augmented feedback facilitates its learning most. Thirty-two able-bodied participants trained grip force with a virtual ball-throwing game for five sessions in a two-week period, using a myoelectric simulator. They received either feedback on movement outcome or on movement execution. Sixteen controls received training that did not focus on force control. Variability over learning was examined with the Tolerance-Noise-Covariation approach, and the transfer of grip force control was assessed in five test-tasks that assessed different aspects of force control in a pretest, a posttest and a retention test. During training performance increased while the variability in performance was decreased, mainly by reduction in noise. Grip force control only improved in the test-tasks that provided information on performance. Starting the training with a task that required low force production showed no transfer of the learned grip force. Feedback on movement execution was detrimental to grip force control, whereas feedback on movement outcome enhanced transfer of grip force control to tasks other than trained. Clinical implications of these results regarding virtual training of grip force control are discussed.

## Introduction

To use an upper limb prosthesis dexterously, one needs training [1]–[3].An evidence-based training should optimally facilitate skill acquisition, thereby enhancing functionality and efficiency with a prosthesis during training, and promoting transfer of skills from training to everyday life situations. Learning to usea prosthesis implies that motor learning takes place, which is generally seen as the permanent changes in behavior as result of practice [4]. Practice is therefore one of the most important factors in motor learning as the degree of improvement depends on the amount of practice [4]–[5]. Another factor that has effect on the motor learning process is feedback [5]. With provision of the correct augmented feedback during or after practice, learning can be maximally enhanced [6]–[7]. In this study, we examined the influence of feedback on the learning process while training with a myoelectric prosthesis. Revealing those motor learning processes of prosthesis users allows designing evidence-based training protocols that optimize these learning processes. Therapists could benefit from such protocols to enhance prosthesis skills.

When one learns a new skill, the performance is characterized with variability at the start that decreases with practice [8]–[11]. The type and degree of variability is an outcome measure that might help us to understand motor learning strategies of prosthesis users. Especially in redundant systems different types of variability can be distinguished [9]–[13]. Redundancy arises when there are more elements than necessary to create an action [8], [12]. For example, the many elements of the human body have numerous degrees of freedom, which results in many different ways in which an action can be performed successfully. Although prostheses have less degrees of freedom than a human arm and hand, this is also the case in prosthesis use. Therefore, studying the change in variability over learning while executing a task with redundancy may provide insight in how certain task solutions (i.e., movements) are chosen from a larger set of possible task solutions. In order to understand how prosthesis users learn to perform certain tasks, it is therefore informative to look at the change in variability over time during learning.

One of the methods to analyze performance in a task with redundancy is the so-called TNC analysis (Tolerance, Noise, Covariation), introduced by Müller and Sternad [10]. They developed a method that divides variability into three different components, Tolerance (T), Noise (N) and Covariation (C). The method not only takes the end result (i.e., the outcome of the performance) into account, but also the execution variables (i.e., how the movement is performed), which is different from most other learning studies that look only at the outcome of performance. In a virtual set-up, Müller and Sternad asked participants to hit a skittle with a ball by controlling two execution variables, angle and speed of the ball at the time of release. Different combinations of the angle and speed resulted in a successful solution, creating redundancy in the task. The end result of the performance was the error of the position of the ball with regard to the skittle. They described the variability in the end result as the sum of the three components, T, N, and C, which all contributed to improvement in the task performance. The task was more tolerant when many adjacent combinations of angle and velocity led to a successful solution. Noise was reflected in the random variation of performance, and covariation showed how various combinations of angle and velocity resulted in the same end result [9]–[10]. In this study, the TNC approach is used to study the learning of grip-force control with myoelectric prostheses. Novice prosthesis users performed a virtual ball-throwing task with a handle that acted as a joystick, which was held by the prosthetic hand. They could control two variables, angle and speed of the ball at the time of release. These variables were controlled by the angle in which the handle was positioned and by the grip force of the prosthetic hand, respectively. Three aspects were investigated with this virtual task. First, the performance over learning was examined by analyzing the variability in performance with the TNC approach. Second, the influence of feedback on performance was examined, and the third aspect that was investigated was the level of grip force control that was learned as a result of the training.

Applying the correct amount of grip force is one of the most difficult aspects in dexterous handling of a prosthetic hand because of the limited intrinsic feedback a prosthesis provides [14]–[17]. Despite many attempts to replace the lost sensory feedback, [see 17–20] artificial feedback is still not applied in commercial available prostheses because its functioning is far from optimal [21]–[23]. The feedback that is available to control actions with a prosthesis is visual information, [24]–[26] which therefore will be the focus in this study. It is known that able-bodied persons can use visual information to prospectively adjust actions to object characteristics [27]–[30]. Despite the limited proprioceptive feedback, a certain level of the control of grip force has also been shown in prosthesis users as well as in studies with neurological patients [18], [20], [31]–[37]. Therefore, we expected that with the provision of the correct type of visual feedback during training, acquisition of grip force control can be optimally facilitated during training, and, more importantly, transfer of the grip force control will be promoted to performance after training. This is of particular importance for a dexterous use of the prosthesis in daily life.

Augmented visual feedback can easily be provided via virtual training systems, which is becoming increasingly popular [38]–[39]. In this study, two types of feedback that are generally used in training, feedback on the outcome and feedback on movement execution, are presented during training in the virtual environment. Feedback on the outcome often leads to improved performance after learning in other tasks than trained [4], [40]–[41]. Feedback on movement execution can lead to better performance during learning, demonstrated in particular in neurological patients [42]. However, some studies show that performance may deteriorate if the feedback is not available anymore after learning [43], indicating that transfer of learning did not take place or was at least suboptimal. It is not known which of these two types of feedback facilitate grip force learning and transfer of the skill; therefore, both types of feedback were examined in the virtual training. Although virtual reality training has shown positive effects on motor learning during training in some studies [44]–[45], to our knowledge there is no systematic study to date that proves learning of prosthetic skills and transfer of those skills to other tasks than trained.

Therefore, the aim of the present study is to determine whether virtual training improves force control in prosthesis use, by examining the variability over learning, and to examine whether virtually provided augmented feedback facilitates learning. We hypothesized that 1) performance will increase during training; 2) variability will decrease over learning; 3) feedback on the outcome will enhance transfer of learning more than feedback on the movement execution; and 4) grip force control will transfer to other tasks than trained.

## Methods

The data collected in this study are available from the authors upon request.

### Participants

Thirty-two able-bodied participants received force control training (11 men, 21 women, mean age (SD) = 21.28 (3.21) years), randomly assigned to either a group that received feedback about the outcome—the landing position of the ball (LF)—or feedback about the movement execution—the applied parameters angle and force, and the trajectory of the ball (TF). Another sixteen able-bodied participants received training that did not focus on force control (CO; 9 males, 7 females; mean age (SD) = 21.56 (2.71) years). All participants were right handed, had normal vision, and had no earlier experience with a myoelectric prosthetic simulator (see Materials).

#### Ethics Statement

The Medical Ethical Committee of the University Medical Center Groningen, the Netherlands (number NL40721.042.12) approved the experiment. Before the start of the experiment, participants signed an informed consent form. They received a gift voucher at the end of the experiment.

### Materials

Participants wore a myoelectric prosthetic simulator to mimic a below-elbow myoelectric prosthesis. The simulator was developed to closely resemble a real prosthesis and was equipped with a MyoHand VariPlus Speed (Otto Bock), with an opening and closing speed between 15–300 mm/s and a grip force control between 0–100 N; both proportionally related to the height of the myoelectric signals). The hand was attached to an open cast in which the hand could be placed that could be attached to the arm using a self-adhesive (Velcro) sleeve. Activation of the extensors of the wrist opened the myoelectric hand whereas flexors closed the hand. See our earlier work [1], [46]–[47] for further details on the prosthetic simulator and the procedure of donning the simulator.

**Figure 1:**
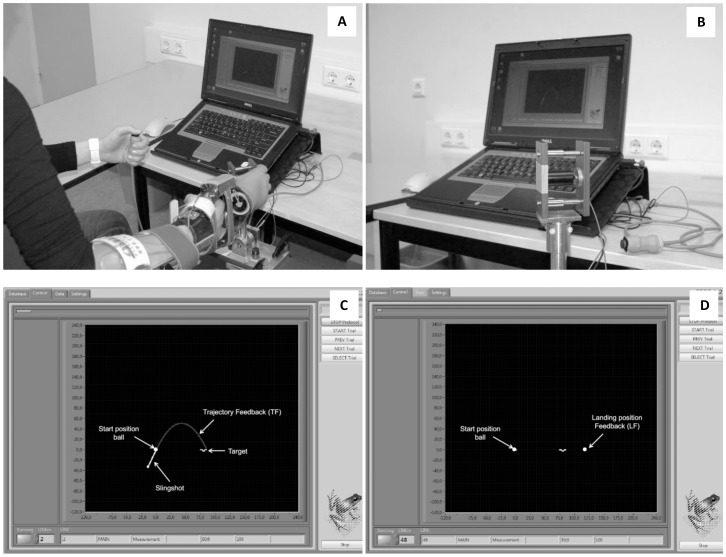
Experimental setup; a participant in action with the prosthetic simulator attached to the right forearm, controlling ball release by pressing a button with the left hand (A), the measurement setup with the handle (B), and two screenshots of a thrown ball with trajectory feedback (C) and with landing position feedback (D).

The experiment was executed with a custom made program on a laptop (created with Labview; display and sample frequency 100 Hz). A handle, comparable with a joystick, was used to execute the tasks (see Figure 1 for the experimental setup). The handle was equipped with a force transducer (LLB350 Loadcell (Futek); maximum force 222 N) and an electrical resistance meter (resistance value ranged from 0KOhm to 10KOhm in an angle from 0–360 degrees) to measure the applied force and the angle of the handle, respectively. The handle could be moved only in one plane, parallel to the table.

**Figure 2:**
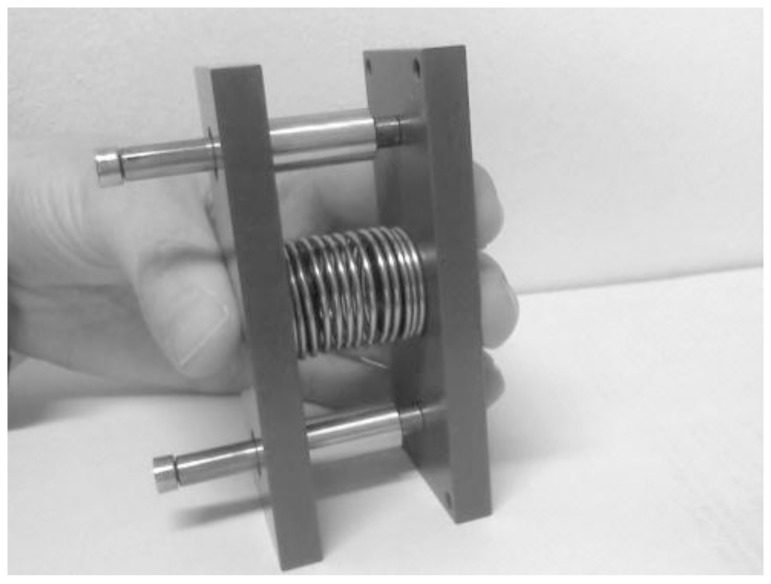
One of the deformable objects consisting of 2 plates with a spring in between.

Three deformable objects were used, consisting of 2 plates (6 cm×3.5 cm×9 cm) with a spring in between (Figure 2), to simulate objects used in daily life such as a milk carton. Each object had a spring with a different constant; a low-resistance object (LO; c = 0.17 N/mm), a moderate-resistance object (MO; c = 0.57 N/mm) and a high-resistance object (HO; c = 5.31 N/mm).

The Box and Blocks Test [48] was used to train the CO group. This physical test allowed participants to practice with the prosthetic simulator without training the force control explicitly. The test was modified for the training purpose, with only 30 blocks instead of 150 and was performed standing instead of sitting because it was easier for the participants to perform the test that way (see also [64]). A laptop with a running stopwatch provided the participants with visual feedback about their performance times.

### Design and procedure

Five test-tasks were assessed that tested different aspects of force control. These test-tasks were applied before (pretest) and after training (posttest) and in a retention test, which was administered two weeks after the posttest. The training consisted of five sessions in which the LF and TF participants trained a virtual force control task, while the CO participants trained with the Box and Blocks Test. The sessions were spread out over a period of two weeks to mimic a rehabilitation setting, in which training is also spread out over a longer period. See Table 1 for an overview of the experimental design.

**Table 1:** Overview of the experimental design.

Session 1				Session 2			Session 3			Session 4			Session 5				Session 6
Day 1 –*Monday*				Day 3 –*Wednesday*			Day 5 –*Friday*			Day 9 –*Tuesday*			Day 11 –*Thursday*				Day 25*–2 weeks after posttest*
Pretest	Training 1			Training 2			Training 3			Training 4			Training 5			Posttest	Retention test
Group	FB	TD	Trials	FB	TD	Trials	FB	TD	Trials	FB	TD	Trials	FB	TD	Trials	Group	Group
LF	LF 20-120	20	75	LF 20-120	80	60	LF 20-120	40	45	LF 20-120	100	30	LF 20-120	60	15	LF	LF
		80	15		40	30		100	45		60	60		120	75		
	LF 120-20	120	75	LF 120-20	60	60	LF 120-20	100	45	LF 120-20	40	30	LF 120-20	80	15		
		60	15		100	30		40	45		80	60		20	75		
TF	TF20-120	20	75	TF 20-120	80	60	TF 20-120	40	45	TF 20-120	100	30	TF 20-120	60	15	TF	TF
		80	15		40	30		100	45		60	60		120	75		
	TF120-20	120	75	TF 120-20	60	60	TF 120-20	100	45	TF 120-20	40	30	TF 120-20	80	15		
		60	15		100	30		40	45		80	60		20	75		
CO	BBTr			BBTr			BBTr			BBTr			BBTr			CO	CO

LF  =  landing position feedback; TF  =  trajectory feedback; CO  =  control; FB  =  feedback; TD  =  target distance.

#### Virtual force training

Participants played a virtual ball throwing game in which they had to throw a ball with a certain angle and velocity into a target by grasping and controlling the handle with the prosthetic simulator. The ball was presented left, attached to a slingshot-spring (c = 1 N/m) that was shown as a white line. The velocity of the ball was determined by the degree of elongation of the slingshot, which in turn was controlled by the force applied to the handle. The more force applied to the handle, the longer the slingshot, with a range of 0-100 N. The angle of ball release was controlled by rotating the handle (range 0 to 90 degrees). After selection of the force and the angle, the ball was released by pressing a button, held in the opposing hand. The ball described a parabolic trajectory (see Appendix S1 for the formulas). Different combinations of angle and force resulted in a hit of the target, which created redundancy in the task.

Six targets were presented during five sessions with 90 trials in each session. The targets differed in x-position on the screen (20, 40, 60, 80, 100, and 120), while the y-position was always zero. Each target was practiced for 75 trials, thereby spreading the trials of the targets over the sessions, resulting in a transition between goals at different times within each session [10] to control for warm-up and retention effects. To control for the influence of target location, the two feedback groups (LF and TF) were split into two subgroups. Participants were randomly assigned to a subgroup that performed tasks in the order of 20-80-40-100-60-120 (LF 20-120 and TF 20-120), the other subgroup performed the tasks in the reverse order (120-60- 100-40- 80-20; LF 120-20 and TF 120-20).

The TF group received feedback about the executed movement: After each trial the elongation and position of the slingshot at the time of ball release and the trajectory of the ball were shown. The LF received only feedback about the end position of the ball.

#### Box and Blocks Training (BBTr)

In each session, participants of the CO group performed the physical, real life BBTr three times. They had to pick up and place 30 objects with the prosthetic simulator from one side to the other as fast as possible, which created a similar motivation as the grip force training group to perform as best as possible. In total, the participants grasped and placed the real blocks 90 times, which is equal to the 90 trials of the experimental group. To provide visual feedback to this group as well, participants received feedback on the movement time, presented with a running stopwatch on a computer screen. Participants self-timed their performance by pushing the spacebar of a keyboard before and after transferring 30 blocks to start and stop the time.

#### Test-tasks

To test the ability of instant force production, the *matching-test task* was assessed. An amount of force was presented on the screen that the participants had to reach in one instant. The requested force (5 to 50 N in steps of 5 N, total of 10 trials in random order) was indicated by an orange marker on the screen. Participants were not allowed to adjust the force once they had produced a certain amount of force.

The *tracking-test task* assessed continuous force control. Participants had to track a pattern for 30 seconds that was displayed on the screen. The course of the pattern, indicated by a yellow line, appeared 200 ms before the red force signal produced by the participant. The yellow line always started with a flat line of 10 N for 3 seconds, after which the pattern started. Three different patterns were assessed; a sine wave, a blocked pattern and a compound sine wave (range of force 0–50 N). Each pattern was executed three times, resulting in 9 trials that were offered in blocked-random order. The range of force used in the matching and tracking test lies within the range needed to carry out activities of daily living [49].

The *picture-test task* was used to assess how well participants could estimate the amount of force they had to apply when seeing a compressible object. Pictures of the MO and HO were shown on the screen, with a certain amount of compression (no compression, half compressed and totally compressed). Participants were instructed to provide the amount of force (to the handle) they thought was needed for lifting and compressing the object in that manner. Before the start of each trial, participants were allowed to experience the objects in real life with the normal hand. Each condition was repeated 2 times, resulting in 12 trials in random order.

The *percentage-test task* tested the ability to estimate the force applied with the prosthetic hand with regard to the maximum. First participants produced their maximum force. After that they had to produce a certain percentage of that force: 25%, 50%, 75%, in random order presented on the screen. Each percentage was repeated 3 times. No feedback was given about the performance.

Next to the four virtual test-tasks that were assessed with the experimental setup, a fifth test was included to assess performance in real life. In the *object-test task* participants had to pick up a compressible object with the prosthetic hand without trying to deform the object. Each object (LO, MO, HO) was assessed 3 times in random order, resulting in 9 trials. A summary of all the five test-tasks is provided in Table 2.

**Table 2:** Summary of the test-tasks.

Task	Short description
Matching test-task	Virtual test-task in which a certain amount of force had to be reached in one instant (5 to 50 N in steps of 5N)
Tracking test-task	Virtual test-task in which a pattern (sine, blocked, or compound sine) had to be tracked for 30 seconds
Picture test-task	Virtual test-task that assessed how well participants could estimate the amount of force they had to apply when seeing a compressible object
Percentage test-task	Virtual test-task in which 25%, 50%, 75%, and 100% of the maximum force had to be produced
Object test-task	Real life test-task in which a compressible object had to be picked up without deformation

### Data analysis

#### Training

The angle, the amount of force produced and the x-coordinate of the end position of the ball were recorded for each trial. These outcome measures were used and analyzed with the TNC approach of Cohen and Sternad [9] using Matlab (Mathworks, R2012), to calculate the costs of the three components of variability, T-cost, N-cost, and C-cost. First, the error—distance to the target—was calculated. See Appendix S1 for the formulas used to calculate the trajectory of the ball and the error. The mean error of five blocks within each target, consisting of 15 trials, was first calculated per participant and then per group. A repeated measures ANOVA was executed on the error with Target (the total number of practiced targets, i.e., number 1 to 6) and block (1 to 5) as within-subject variables and Feedback (LF and TF) and Target order (20-120 and 120-20) as between-subject variables. To examine the performance of the different groups during training, three different repeated measures ANOVA's were executed on the three variables, T-cost, N-cost, and C-cost, with Target (number 1 to 6) and Block (1 to 5) as within-subject variables and Feedback (LF and TF) and Target Order (20-120 and 120-20) as between-subject variables.

Mean time and standard deviation of performance on the BBTr was calculated over all control participants for each of the trials in the five sessions.

#### Test-tasks

Error was defined as the difference between the produced force and the asked force, and a mean deviation was calculated between the produced force and the asked force for the four virtual tests and the error was defined as the amount of compression in the object test. Five separate repeated measures ANOVAs were executed on the error with Feedback (LF, TF, and no feedback) and Target Order (20-120, 120-20, and control group) as between-subjects factor and Test (pretest, posttest and retention test) as within-subjects factor for all test-tasks and Condition as within-subjects factor for four of the five test-tasks. The matching task had no different conditions; the tracking task had three conditions (sine wave, blocked pattern, and compound sine wave); the picture task had six conditions (no compression MO, MO half compressed, MO totally compressed; and no compression HO, HO half compressed, HO totally compressed); and the percentage task had three conditions (25%, 50%, and 75%). After examining the data, the two no-compression conditions of the picture task were removed from the analysis because results on these conditions were not accurate as the applied force was sometimes not measured by the force transducer. Although the instruction was to produce the amount of force needed to lift the object without compression, some of the participants only applied less force than was minimal required to register the force with the force transducer. Therefore the results were too variable to analyze. Moreover, for the object test, only data from 16 participants were analyzed (only the LF 120-20 and the TF 120-20 groups), because the data of the other participants was not collected correctly.

All analyses used a significant criterion of α = .01 because of the large number of tests performed, and post hoc tests on main effects used Bonferroni adjustment. In case of violation of sphericity in Mauchly's test the degrees of freedom were adjusted with the Greenhouse-Geisser correction.

## Results

### Virtual training

**Figure 3:**
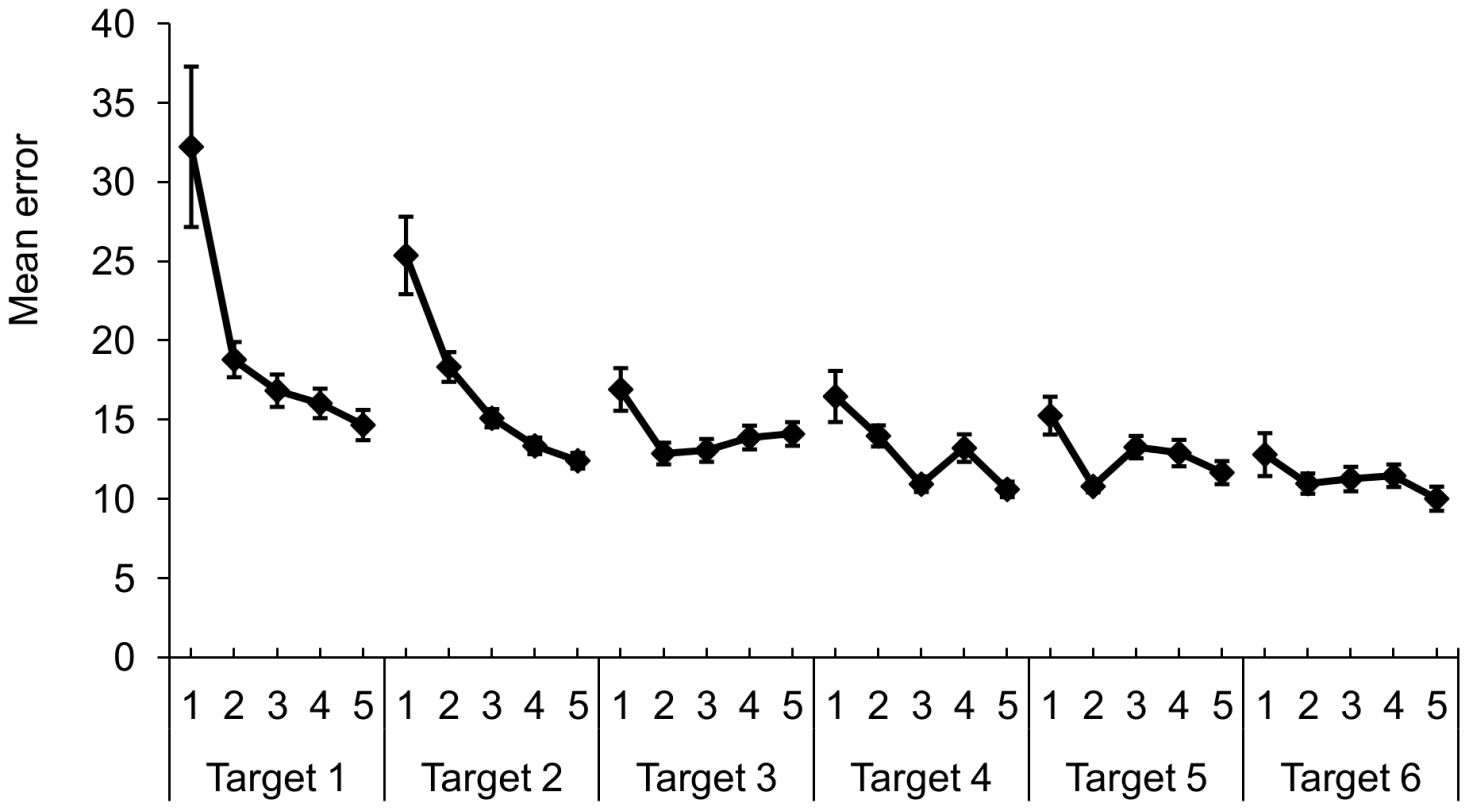
Mean error (SE) across participants over targets that were presented to the participants and the five blocks of 15 trials within each target.

An overall decrease of error (Figure 3) was seen with practice over the number of targets and over the blocks within each target (Table 3). The largest decrease occurred at the beginning of the training period and at the start of each new target, especially in the first two targets presented (small interaction effect of target x block F (4.99, 139.66) = 5.45; *p* = .00; η^2^_G_ = .04). No main effect of feedback was found.

**Table 3:** Statistics of main effects with means and standard errors(SE) in the virtual training for the overall Error during training and the three components Tolerance, Noise and Covariation.

Dependent variable	Factor	Condition	Mean (SE)	F	df	*P*	η^2^_G_	95% CI
Error	Target	1	19.93 (1.81)	12.80	1.87, 52.45	.00	.07	[16.22, 23.64]
		2	16.91 (1.52)					[13.80, 20.01]
		3	14.16 (1.90)					[12.31, 16.01]
		4	13.03 (.72)					[11.56, 14.50]
		5	12.77 (.69)					[11.36, 14.18]
		6	11.30 (.60)					[10.07, 12.52]
	Block	1	19.84 (1.45)	31.34	2.14, 59.78	.00	.05	[16.87, 22.80]
		2	14.28 (.72)					[12.80, 15.76]
		3	13.40 (.68)					[12.02, 14.79]
		4	13.47 (.84)					[11.75, 15.18]
		5	12.42 (.70)					[10.98, 13.87]
Tolerance cost	Target	1	4.76 (.25)	6.93	2.50, 42.49	.00	.06	[4.24, 5.29]
		2	5.03 (.23)					[4.55, 5.51]
		3	5.08 (.22)					[4.61, 5.55]
		4	5.53 (.26)					[4.98, 6.09]
		5	5.52 (.20)					[5.10, 5.95]
		6	5.71 (.26)					[5.15, 6.26]
Noise cost	Target	1	11.18 (1.09)	6.35	2.39,66.68	.00	.02	[8.94, 13.41]
		2	10.04 (.69)					[8.62, 11.45]
		3	9.04 (.64)					[7.72, 10.36]
		4	8.25 (.60)					[7.02, 9.48]
		5	8.09 (.55)					[6.98, 9.21]
		6	7.80 (.52)					[6.74, 8.86]
	Block	1	10.98 (.74)	8.91	2.94, 82.56	.00	.02	[9.47, 12.48]
		2	8.91 (.64)					[7.61, 10.22]
		3	9.03 (.60)					[7.80, 10.26]
		4	8.38 (.62)					[7.11, 9.64]
		5	8.03 (.53)					[6.95, 9.10]
Covariation cost	Block	1	3.59 (.67)	9.47	2.58,72.28	.00	.02	[2.23, 4.95]
		2	2.62 (.58)					[1.44, 3.81]
		3	2.32 (.55)					[1.20, 3.44]
		4	2.34 (.56)					[1.20, 3.49]
		5	2.08 (.42)					[1.22, 2.93]

CI  =  confidence interval.

**Figure 4:**
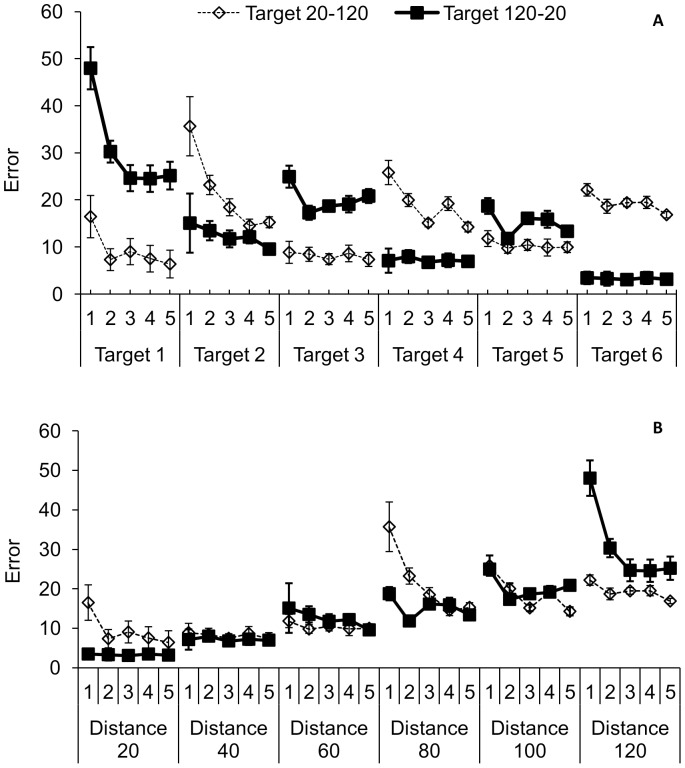
Performance error (SE) of the participants for each of the five blocks of 15 trials in the practiced targets (target 1 to target 6) for both groups that practiced in the order 20-80-40-100-60-120 and 120-60-100-40-80-20 (4A) and the error plotted against each of the target distances (4B) for each of the five blocks of 15 trials within each target distance.

A large interaction effect of target x target order (F (1.87, 52.45) = 69.03; *p* = .00; η^2^_G_ = .38; Figure 4A) showed that the distance of the target influenced the amount of error, with a larger distance resulting in more error. Therefore, the two groups that practiced the targets in reverse order differed largely in the error at each target. When comparing the error for each of the target distances (Figure 4B), it can be seen that relatively the most error is made in the target distance that was started with. The last target distance that was practiced had the least error.

### Use of the execution variables force and angle

**Figure 5:**
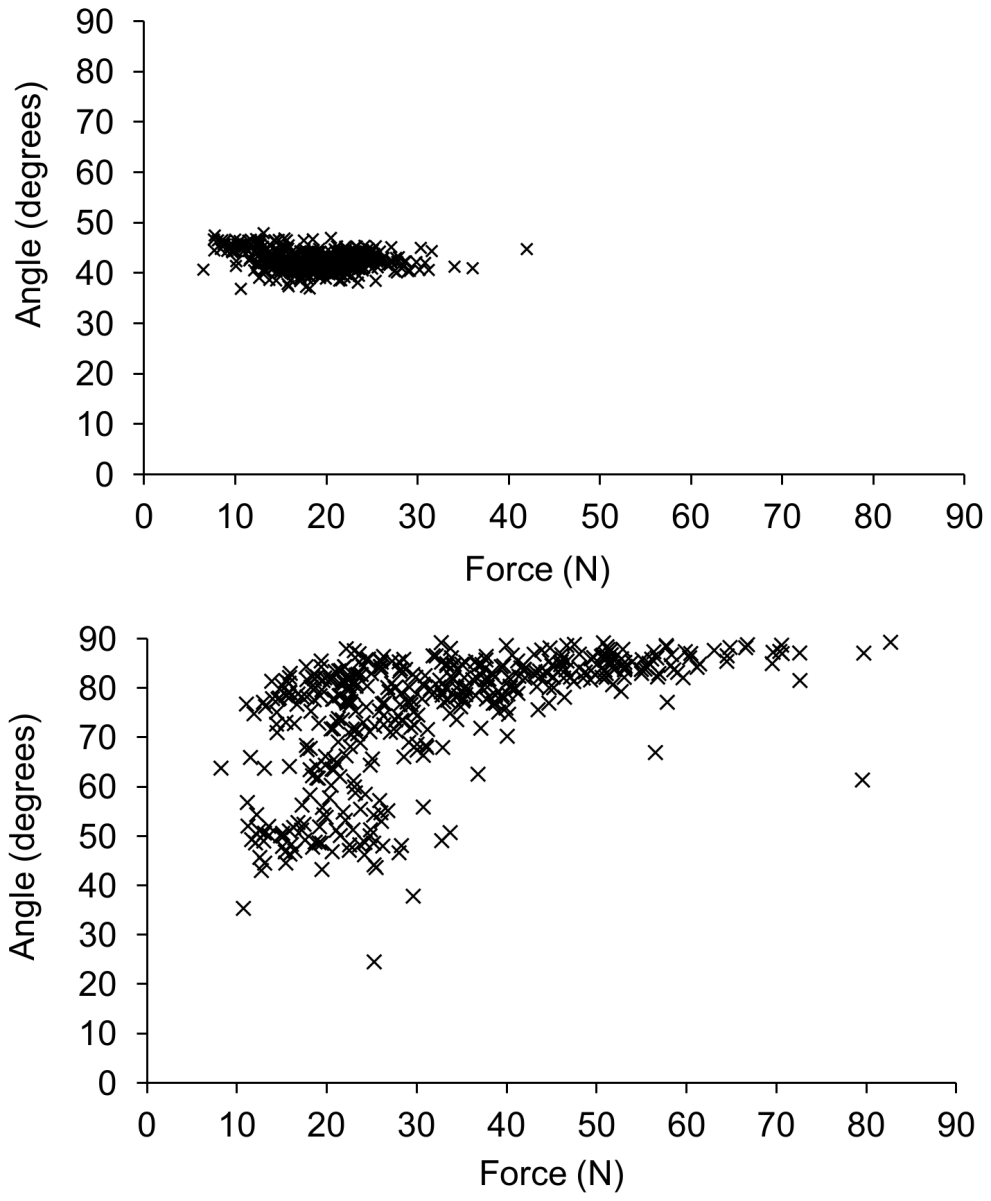
Two typical examples of the strategies can be seen. One strategy was to hold the angle constant while varying the force (A), the other strategy was to vary both angle and force (B). For each of the strategies, all trials of a typical participant were plotted over sessions and over targets. Each data-point represents a trial.

**Figure 6:**
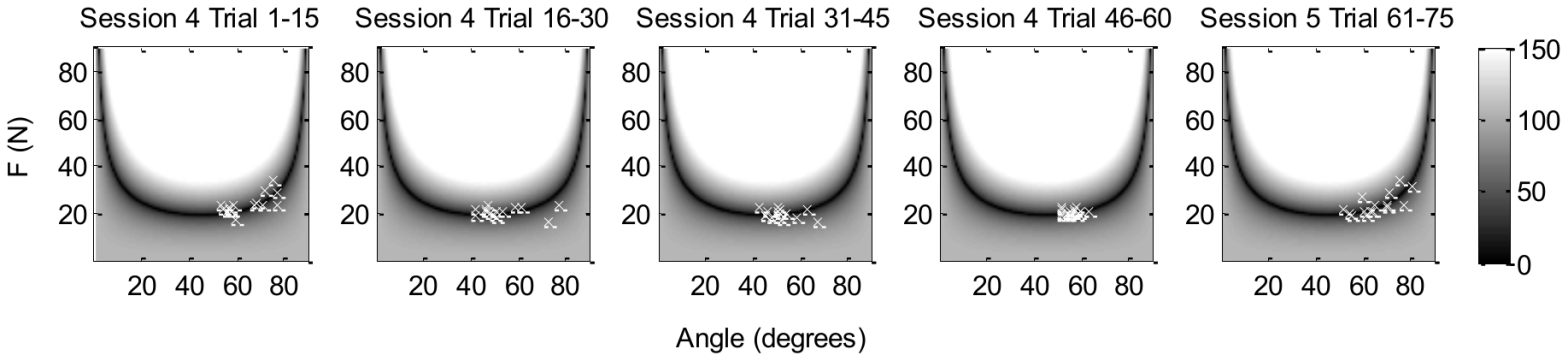
Forces and angles produced by one of the participants from the TF 120-20 group are plotted against each other for all trials of the target with distance 80. Each panel represents 15 trials. During practicing a decrease in variation of data points can be seen over the first four plots, which shows improvement during practice within the target. From session 4 to session 5, thus from the fourth to the fifth plot, a deterioration in performance can be seen, possibly due to increased exploration of the solution space. The shades of grey represent the distance of the ball with regard to the target.

The mean applied force was 20.01 N (median 19.35 N, IQR = 3.42 N), although the range of applied forces was large; 4 N to 90 N. The mean angle used concentrated around 50 degrees (median 49.62, IQR = 16.36 degrees), with a range from 15 to 88 degrees. Two different strategies were noticed during the training and while examining the data. One strategy was to hold the angle constant while varying the force (12 participants with LF and 6 participants with TF); the other strategy was to vary both angle and force (4 participants with LF and 10 participants with TF; see Figure 5 for typical examples). Figure 6 shows an example of the performance over time in solution space plots. Notice the decrease of variability over trials within session 4, while the spread in error is larger again when the next trials of that target are practiced in a subsequent session. This might be due to temporary increased exploration around the earlier found solution.

### Variability measures

**Figure 7:**
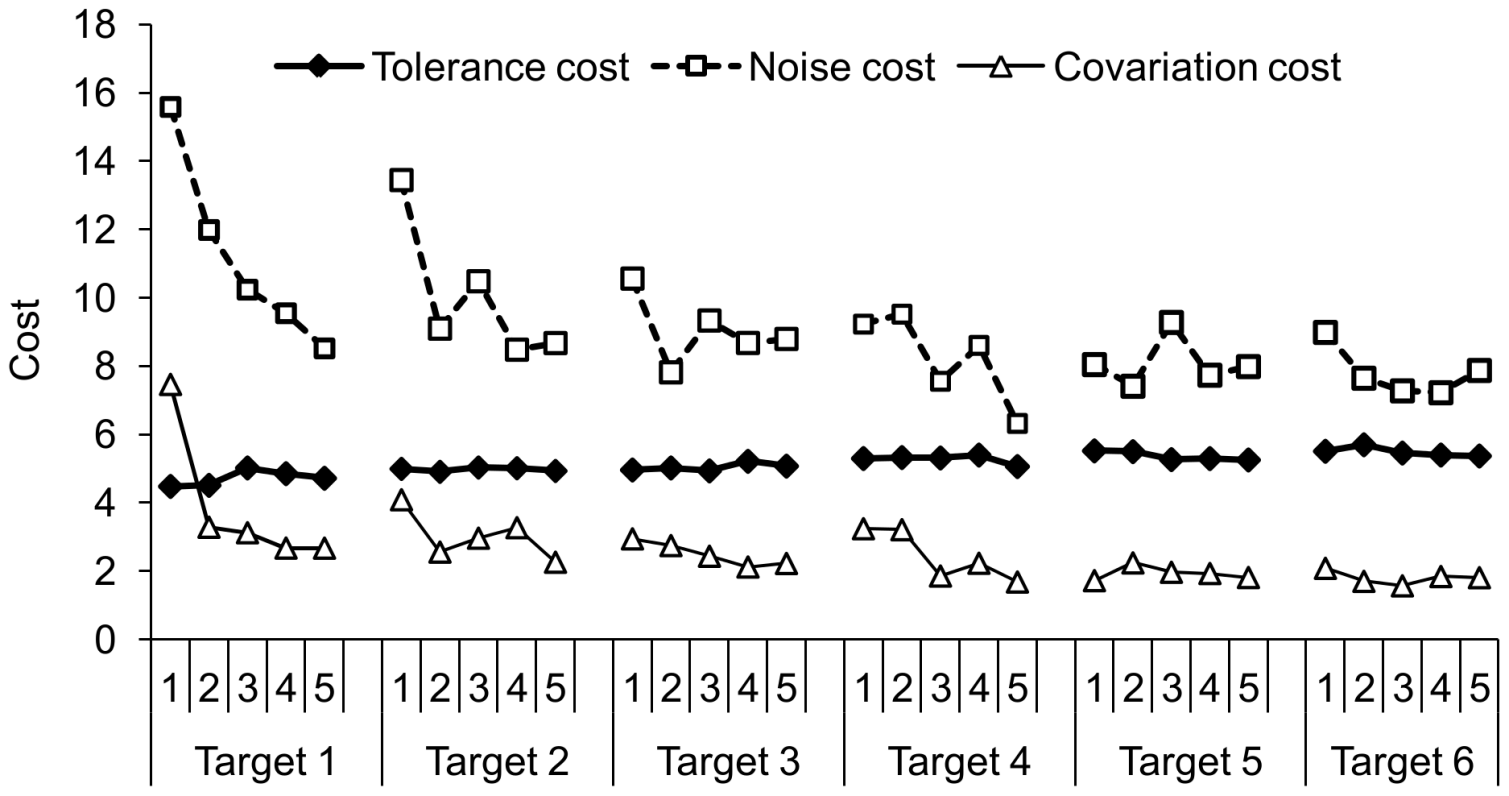
The progress of T-cost, N-cost, and C-cost over the number of targets practiced and over the blocks of 15 trials within the targets.

*T-Cost* increased slightly over the number of targets performed (Table 3). The T-cost was not affected by location of the target, nor did the type of feedback result in significant differences. *N-cost* was higher for larger target distances, but decreased overall (Table 3). Within most of the targets the noise decreased as well (Figure 7). A large target x target order interaction (F (2.39, 66.86) = 89.77, *p* = .00; η^2^_G_ = .36) showed that, similar to the overall error, the error was different for the different target distances, and as the 20-120 and the 120-20 groups practiced targets in reverse order, this resulted in a large difference in noise. Type of feedback did not affect N-cost. A small main effect of block revealed that *C-cost* decreased over blocks within each target (Table 3), and a trend in decrease of C-cost was seen over the number of targets (F (1.75, 48.99) = 3.33, *p* = .05, η^2^_G_ = .03). A small interaction effect of target x block (F (5.77, 161.45) = 3.92, *p* = .00; η^2^_G_ = .02) revealed that the C-cost decreased mainly from block 1 to block 2 within the first two targets. A small target x target order interaction (F (1.75, 57.69) = 6.10, *p* = .01; η^2^_G_ = .02) showed that the C-cost differed for the 120-20 and the 20-120 groups over targets, just as the noise and the overall error.

### Box and Blocks training

**Figure 8:**
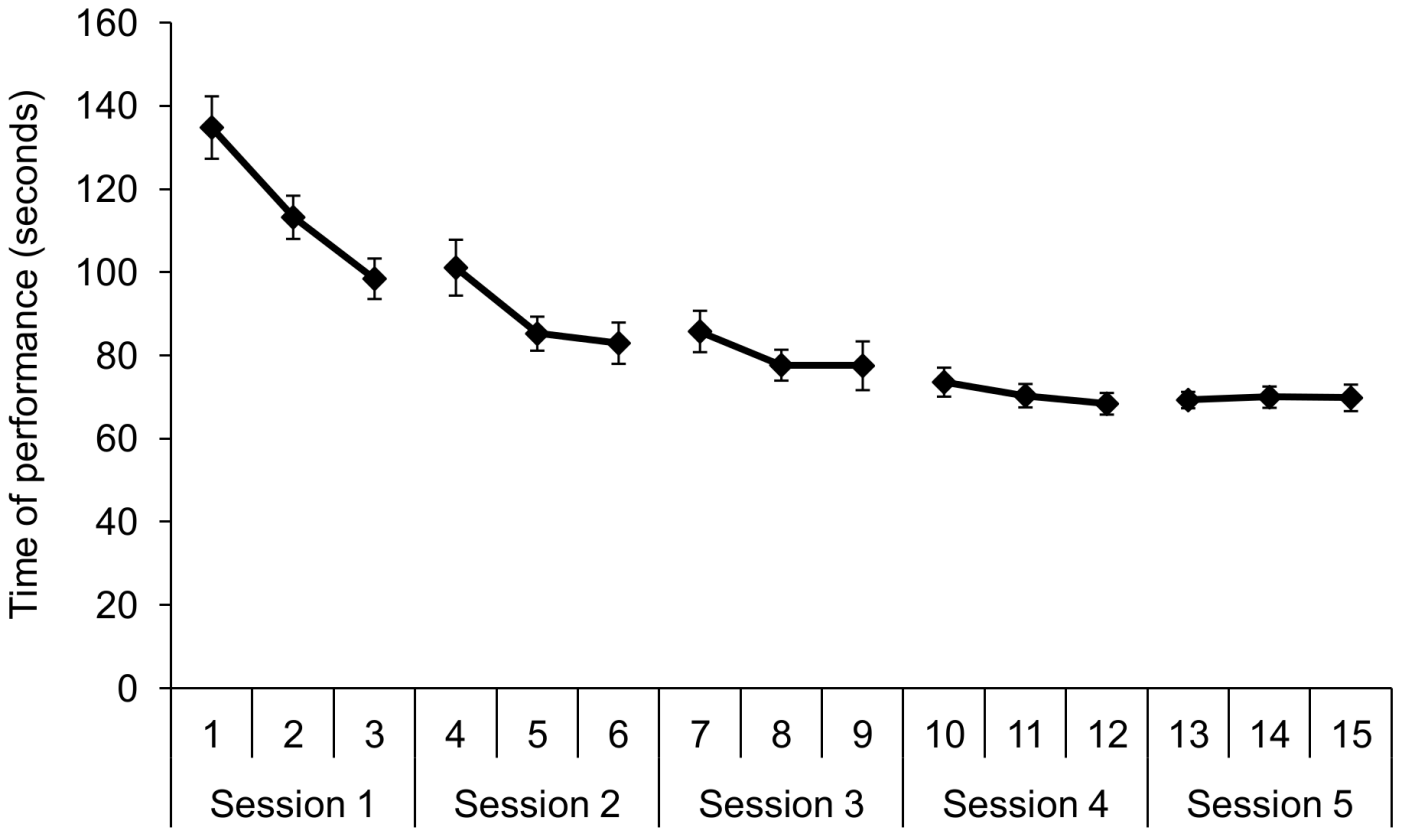
Mean (SE) for the performance on the Box and Blocks training in which 30 blocks had to be transferred from one side of the box to the other.

Participants in the CO group improved their performance time over the sessions from a mean score of 134 seconds to 69 seconds. In the first training sessions, time of performance decreased quickly, while later on in training the improvement slowed down (Figure 8).

### Test-tasks

#### Matching test-task

Main effect of test showed that participants improved in the posttest compared to the pretest, however, their improvement did not last in the retention test (Table 4). A small to moderate test x target order interaction (F (2, 84) = 13.23, *p* = .00; η^2^_G_ = .09) showed that the deterioration from the posttest to the retention test was mainly due to the 20-120 group (Table 4).

**Table 4:** Significant effects in the test-tasks with means and standard errors(SE).

Dependent variable	Factor	Condition	Mean (SE)	F	Df	*P*	η^2^_G_	95% CI
Matching test	Test	Pretest	14.46 (.90)	7.30	2,84	.00	.05	[12.65, 16.28]
		Posttest	11.72 (.87)					[9.98, 13.47]
		Retention test	15.04 (.77)					[13.49, 16.60]
	Test x Target Order	20-120 - Pretest	12.34 (1.49)	13.23	2,84	.00	.09	[9.33, 15.36]
		20-120 - Posttest	11.48 (1.44)					[8.58, 14.38]
		20-120 - Retention test	18.84 (1.28)					[16.27, 21.42]
		120-20 - Pretest	16.89 (1.48)					[13.88, 19.91]
		120-20 - Posttest	12.00 (1.44)					[9.10, 14.90]
		120-20 - Retention test	12.85 (1.27)					[10.27, 15.42]
		CO - Pretest	13.85 (1.54)					[10.73, 16.96]
		CO - Posttest	11.64 (1.48)					[8.64, 14.62]
		CO - Retention test	11.85 (1.32)					[9.19, 14.51]
Picture test	Condition	MO half	49.05 (2.95)	37.50	2.05, 86.02	.00	.20	[43.10, 55.00]
		MO total	41.90 (1.80)					[38.28, 45.53]
		HO half	23.16 (1.01)					[21.12, 25.20]
		HO total	49.23 (1.47)					[46.26, 52.20]
	Target Order	20-120	44.03 (1.38)	16.02	1,42	.00	.02	[41.26, 46.81]
		120-20	36.11 (1.42)					[33.23, 38.98]
		Control	43.90 (1.38)					[41.13, 46.68]
Tracking test	Test	Pretest	11.24 (.69)	20.35	1.55, 66.61	.00	.09	[9.90, 12.60]
		Posttest	8.61 (.38)					[7.84, 9.37]
		Retention test	8.87 (.25)					[8.37, 9.38]
	Condition	Sine	10.36 (.44)	15.59	1.75, 75.25	.00	.02	[9.48, 11.24]
		Blocked	9.54 (.37)					[8.79, 10.28]
		Complex sine	8.83 (.41)					[8.01, 9.65]
Percentage test	Condition	25%	30.95 (2.40)	40.49	1.75, 71.63	.00	.21	[26.10, 35.79]
		50%	31.60 (1.26)					[29.06, 34.14]
		75%	19.89 (.85)					[18.17, 21.21]
Object test	Test	Pretest	9.66 (.83)	4.62	2, 56	.01	.03	[7.97, 11.36]
		Posttest	8.30 (.88)					[6.51, 10.09]
		Retention test	7.15 (.83)					[5.46, 8.85]
	Condition	LO	13.75 (1.02)	174.60	1.56, 43.54	.00	.51	[11.67, 15.83]
		MO	11.27 (.99)					[9.24, 13.29]
		HO	0.10 (.06)					[.002, .219]
	Condition x Target Order	120-20 – LO	12.18 (.91)	6.13	1.56, 43.54	.01	.04	[10.31, 14.05]
		120-20 – MO	8.50 (.89)					[6.69, 10.32]
		120-20 – HO	0.14 (.05)					[.030, .248]
		CO – LO	15.32 (1.82)					[11.60, 19.04]
		CO – MO	14.03 (1.77)					[10.41, 17.65]
		CO – HO	0.06 (.11)					[.016, .273]

CO: control group; LO: Low-resistance Object; MO: Moderate-resistance Object; HO: High-resistance Object; CI  =  confidence interval.

#### Tracking test-task

**Figure 9:**
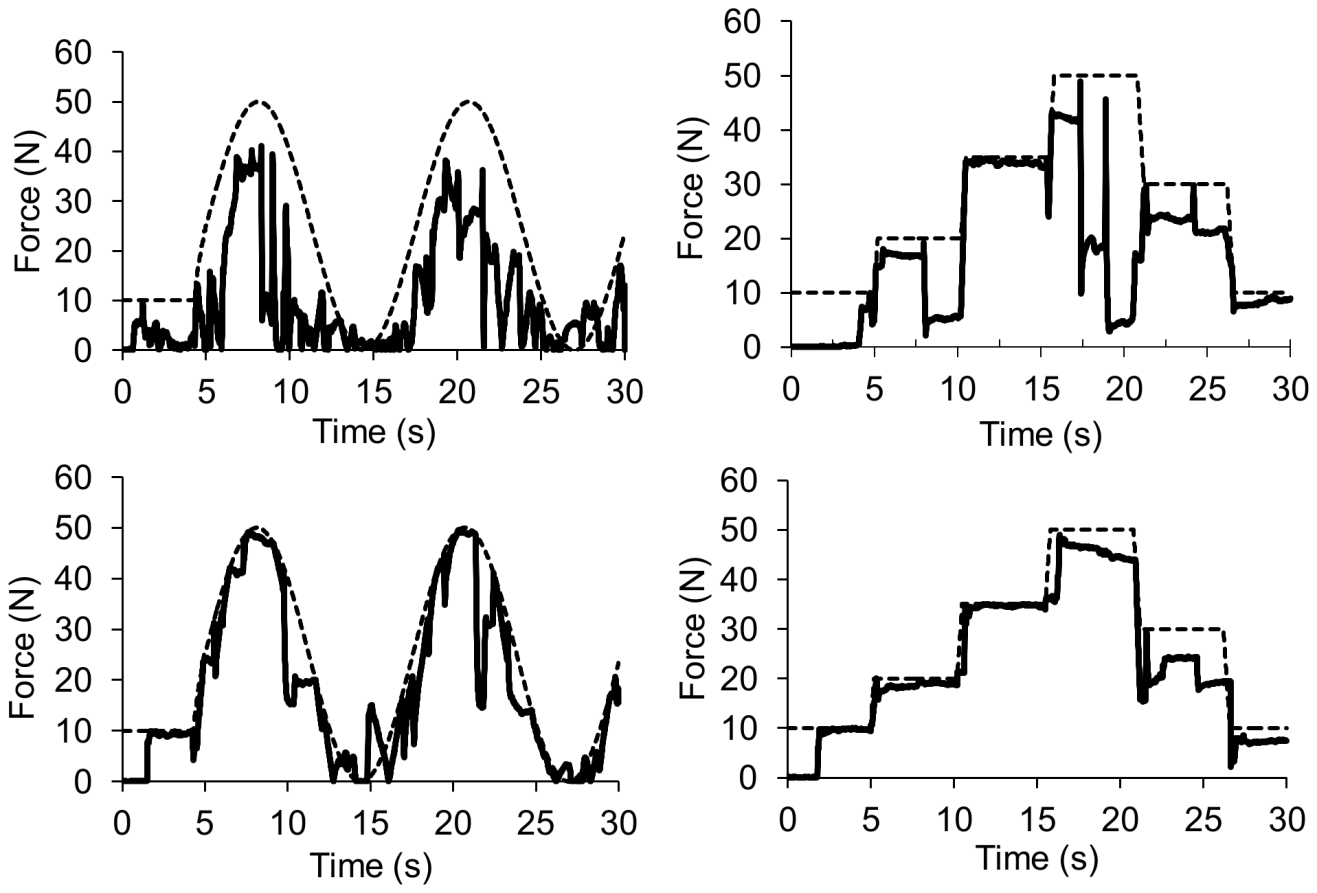
Performance of an arbitrary selected participant of the simple sine pattern and the blocked pattern during the pretest (above) and the retention test (below). The dashed line represents thepattern asked by the computer, the performance of the participant is shown with the thick line. Increasing the applied grip force was easier to control than letting go, shown by larger drops in the signal. This performance was seen in many participants.

Participants improved from pretest to posttest in the tracking test-task, and performed on the same level in the retention test (Table 4). Figure 9 shows typical examples of performance in the pretest and the retention test for the sine pattern and the blocked pattern. The compound sine pattern was executed with the least amount of error, while the most error was made on the simple sine pattern, shown in a small main effect of condition. A small main effect of target order showed that the CO group and the 120-20 group had significantly less error than the 20-120, revealed with pairwise comparison (*p'*s<.01).

#### Picture test-task

No significant difference between the three tests (*p* = .06) was found. A moderate main effect of condition showed that participants had the least error on the HO half compressed condition, which differed significantly from the other three conditions in pairwise comparison (Table 4). A small main effect of target order showed that the 120-20 had significantly less error than the 20-120 and CO groups. A moderate interaction effect of condition x target order showed that the 20-120 and the CO groups had the same errors on the four conditions, while the 120-20 had more error on the MO half compressed condition, but performed much better on the MO completely compressed condition.

#### Percentage test-task

A moderate main effect of conditions was found; the 75% differed significantly from the 25% and 50% of maximal force conditions, shown by pairwise comparison (both *p* = .00). Participants were more capable to estimate 75% of their maximum force than 25% and 50%. No other effects reached significance.

#### Object test-task

A main effect of test showed that performance improved from pretest to posttest and retention test, with a significant difference shown between pretest and retention test, indicated by pairwise comparison (Table 4). The amount of compression differed largely per object; the object with the highest resistance (HO) was almost not compressed while the most compression occurred in the object with the least resistance (LO) (Table 4). A condition x target order interaction (F (1.55, 43.54) = 6.13, *p* = .01; η^2^_G_ = .04) revealed that the participants that trained with the 120-20 order compressed the LO and MO objects less than the controls (Table 4).

## Discussion

### Performance during the virtual force control training

The participants decreased their error over the five sessions and within each of the presented targets, which confirms the first hypothesis that was stated in the introduction. This showed that the participants improved their performance over the training sessions and thus, that they were able to learn to improve their control over practicing the virtual training using visual feedback. The type of feedback did not influence the improvement during training, nor did the order in which the targets were presented. Relatively the most error was made on the first target that was presented and the least amount of error on the last target in the fifth session. The higher error scores for larger target distances was inherent to the design of the task. A shift of 1 degree in the slingshot angle resulted in a small change in the ball's landing position when shooting at a nearby target while in case of a target further away a small change in the angle substantially could affect the landing position.

### Analysis of variability over learning

To test the second hypothesis, variability in performance was decomposed into the three components T-cost, N-cost, and C-cost, according to the TNC approach [9]–[10]. This enabled us to examine what elements contributed the most to the reduction of the error. T-cost did not show large changes during the training period, and therefore did not contribute to improvement. This could be due to the location of the targets used during training. The position of the targets only varied in horizontal direction, which made the solution space of all targets rather alike. It could therefore be that participants found a stable region in the first target and were not challenged to exploit the solution space when new targets had to be reached [10]. The N-cost contributed the most to error in performance, because N-cost was mainly reduced over the training sessions. This finding is in line with the results reported by Cohen and Sternad [9]. When a new target was presented the N-cost increased after which it reduced quickly again over trials. It is likely that participants sought new good solutions by finding new combinations of angle and force [10]. This increased the noise component of variability and, furthermore, the C-cost at the start of a new target, which is what we found.

C-cost was rather small and decreased quickly within each target, as participants anticipated quickly on a change in target location. When a target appeared that was farther away than the previous target, they immediately elongated the slingshot by applying more force to the handle compared to the previous target, and vice versa. This showed that participants anticipated to changes in the demands of the task, and were able to use covariation of the two execution variables to find new successful solutions. The majority of the participants were more inclined to vary the force than the angle when targets changed. They chose mainly angles in the midrange, with the handle pointing upwards, thus, avoiding angles in which they had to position their prosthesis in an awkward posture. In conclusion, to confirm the second hypothesis the variability in performance decreased over practice. This was mainly because of a reduction in N-cost which suggests that within their found movement strategy the participants learned to increase their accuracy by reducing the random fluctuations in performance.

### Influence of feedback on performance

No main effect of feedback was seen during training, although the type of feedback seemed to influence the strategy used. Feedback about the trajectory seemed to elicit more combinations of different angles and forces, while feedback about the landing position resulted often in a strategy to restrain the variations in the angle in order to find a good solution by only varying the force component. The different strategies were not reflected in the C-cost component of the TNC analysis though, which might indicate that both types of feedback were equally effective to manage the covariation of the two variables and to perform equally during training. Because both strategies ensured the continuous practice of the force component (see Figure 5), it can be assumed that the virtual training with visual feedback that is used in this study is suitable for practicing the grip force control.

The type of feedback provided during training did seem to influence the transfer of the learned grip force control to the tests. Although the effect of feedback did not reach the significance level of p = .01, trends were seen in the data. The near significant effects of feedback on the matching test-task (p = .04), tracking test-task (p = .03), and the object test-task (p = .08), and the near significant interaction of feedback by test in the matching test-task (p = .02) and the picture test task (p = .02) showed that the feedback on movement outcome (LF) enhanced transfer of the learned skill more than feedback on movement execution (TF). The LF group improved more from pretest to posttest, and scored overall better on the retention tests than the TF group.

An explanation for the better performance of the LF group could be found in the amount of information provided to the learners during training. Whereas the LF group only received information on the end position of the ball, the TF group received all the information that was available, including the applied force and angle represented by the slingshot and the ball trajectory. According to the guidance hypothesis [41]–[42], provision of too much information is detrimental to learning as learners become reliant on the provision of feedback. This does not challenge people to find solutions on their own, while learners are encouraged to actively search for solutions to the problem when less information is available [52]. Moreover, motor planning is believed to be executed in terms of end-effector space [42]. Therefore, actions may be more effective if they are planned in terms of their outcome rather than in terms of the specific movement patterns.

It might be that the LF group learned to actively plan their movements in terms of their outcome, as well as the CO group who achieved similar performances. They could have developed successful solutions based on other information that they found useful during learning. A small part of the proprioceptive information is still present in prosthesis use, and informs the wearer about the degree of contraction of the muscle. As the degree of muscle contraction was coupled to the velocity of opening and closing of a prosthetic hand, they might have been able to match the degree of contraction to the result of performance. The participants in the LF virtual group received visual information regarding the end result, whereas the CO group could have learned the scaling of muscle contraction too as they were challenged in an accuracy-velocity trade off to perform as quickly as possible. The TF group, however, might have been unable to pick up this little part of information as it was overruled by the provision of too much visual information [53]. Thus, results in this study show that practicing with more feedback does not always seem to be beneficial to skill learning with a prosthesis, which supports the third hypothesis. It might therefore be suggested better to provide information on just the outcome of the movement during training.

### Improvement in grip force control

To test the fourth hypothesis, grip force control was assessed with five test-tasks that concerned different aspects of the control. In the matching and the tracking test-tasks, which are previously used when assessing grip force [31], [54]–[56] performance improved from pretest to posttest. Performance on the two estimation test-tasks, the percentage and the picture test-task, was highly variable between and within participants, and did not show improvement after training. Earlier studies have also shown that performance with a prosthesis is more consistent and less variable with visual feedback than without the provision of information [18], [24]. The performance on the task that assessed grip force with real objects instead of virtually, did improve from pretest to retention test. This is an important result because it shows that transfer of learning can occur from this virtual training to a real life task.

The improvements that were seen in performance after training were not very large, while in one test the improvement did not last as performance decreased again in the retention test. It could be that the training was too short to achieve major improvements and consolidation. In an earlier study it has already been shown that improvement in grip force control requires a lot of time [1]. The current study supports these earlier findings; Grip force control needs to be practiced over a long period

Transfer of the learned grip force occurred in the test-tasks that provided instant feedback about performance, while no transfer was seen in the test-tasks that required estimation of the applied grip forces. According to the specificity of practice hypothesis [57], transfer of learning is most effective when the test resembles the training as closely as possible [40]. It is believed that motor learning and skill enhancement improve the most when similar sources of information are available during training and testing [58]. This could explain why transfer did not occur in the estimation tests. The information provided during training and the matching and tracking test-tasks was rather similar as the learners received concurrent feedback on the applied force, either with a change in the elongation of the slingshot or with a change in the signal that represented the applied force in the test-tasks. In the object test-task the participants were able to notice the compression of the object, which provided them with information as well. The estimation tests, on the other hand, did not provide any information about the performance and did not have any similarities with the training. It might therefore be that because the participants did not practice to estimate their applied force, transfer to the estimation tasks did not occur. Thus, to enhance grip force control learning the most, it might be suggested to include the practice of estimating the applied force as well, besides training grip force with feedback to cover all aspects of grip force control during training.

Another factor that influenced the transfer of learning was the order of target presentation during training. The participants who practiced in the target distance order 20-120 performed poorer on the tests than the participants who trained the 120-20 order. The difference between the two target distance orders is that the 20-120 group started with the 20 target which required low forces to be produced in the beginning, whereas the 120-20 group started with the 120 target that allowed for larger forces. As it is more difficult to produce low forces, especially when starting to learn force control [1], [3], [33], we might conclude that starting with a target in which more force is allowed leads to better performance after training than starting with a target in which low forces are required. We therefore recommend to start practicing with easy tasks that allow for high force productions.

### Training in virtual reality

The results showed that the virtual training of the LF group was as effective as the functional training, executed by the controls, while the TF group performed poorer after training. Thus, although virtual training seems like a useful method in the rehabilitation process [59], this study shows that one should carefully design a virtual training in order to achieve improved performance and transfer of the learned skills to other tasks than trained. The task that needs to be practiced, the amount and the type of information that is provided, and the difficulty of the training are all aspects that seem to influence the learning process in virtual training.

This study only addressed grip force control in an isolated laboratory setting. What remains to be proven is the transfer of skills when using the prosthesis in everyday life. Is it possible to generalize the skills learned during virtual reality to daily practice? While some studies have already shown that control of the myoelectric signals can be learned virtually [see 60 for a review], the effectiveness of the virtual training to improve handling the prosthesis in surrounding space and during manipulation of objects needs to be studied in large randomized controlled studies [60]–[61].Questions are raised whether the transfer of skill can be made from the virtual environment to the real world, since the visual space (i.e., the screen)is not aligned with the workspace of the movements (i.e., the end-effector such as the hand)[62]–[63]. Sensorimotor transformations need to be learned to map the movements displayed virtually with the movements made with the end-effector [62]–[63]. The kind of information and the amount of information that is perceived during virtual training plays a role in this issue. In the object test-task, which mimicked an everyday activity the most, improvements were seen for the virtual training group. This may indicate that it is possible to transfer the skill learned during virtual training to more functional tasks.

### Limitations of the study

A limitation of the study is the design of the virtual task used in this study. Shooting at larger target distances automatically resulted in higher errors. In order to get a clearer picture on the amount of error made in each target, the task should be designed differently. Moreover, the locations of the targets did not vary in y-position, which resulted in rather similar solution spaces. A future study should include variation in y-position as well in order to challenge the participants to exploit the solution space more. Another limitation of this study is the use of a prosthetic simulator instead of real amputees. Because of the limited number of novice prosthesis users, we chose to study the grip force learning processes with a prosthetic simulator that allowed for inclusion of more participants. Although it is not yet known whether the results can be generalized to the population of prosthesis users, an earlier study with the use of the prosthetic simulator showed comparable scores on a functional test and movement characteristics with real prostheses [1].

## Conclusions

Performance increased during virtual training of force control with a prosthetic simulator, reflected in a reduction in error. Using the TNC approach, variability was shown to decrease mainly as a result of the reduction of N-cost and a good covariation between the used force and angle during training. Grip force control improved only in the test-tasks that provided information on the performance. Starting the training with a task that required low force production decreased transfer of the learned grip force. Whereas feedback on movement execution was detrimental, feedback on the movement outcome enhanced transfer of the grip force to other tasks than trained.

## Supporting Information

Appendix S1 **Formulas used for calculations.**
(DOC)
