# Supplementary material for: Effect of Feedback during Virtual Training of Grip Force Control with a Myoelectric Prosthesis
Source: PLoS One. 2014 May 27;9(5):e98301. doi: 10.1371/journal.pone.0098301 (PMC4035328; doi:10.1371/journal.pone.0098301)
Supplement: Appendix S1 — Formulas used for calculations. (DOC) [file pone.0098301.s001.doc]

Appendix S1. Formulas used for calculations

The following formulas were used to determine for the produced angles (alpha) and forces (F) the parabolic trajectory of the ball- given spring constant c and the balls’ mass m. The travelled distance was calculated and the distance between target and ball landing (error).

c = 1.0 N/m

m = 0.5 kg

g = 9.81 m/s2

| Kinetic energy: | Ek = 0.5 * m * v^2^ = Ev = 0.5 * F^2^ /c |
| --- | --- |
| Initial velocity of the ball: | v_i_ = F / √(m * c) |
| y-component of the initial velocity: | y(t) = vi_y * t - 0.5 *g * t^2^ |
| x-component of the initial velocity: | x(t) = vi_x * t |
| Ground hit of the object (y(t) = 0): | t_g = 2 * vi_y / g |
| Distance traveled at moment of ground hit: | x(t_g) = vi_x * t_g = 2 * vi_y * vi_x /g |
|  | vi_y = v_i_ * sin (alpha) |
|  | vi_x = v_i_ * cos (alpha) |
|  | x(t_g) = vi^2^ * sin(2 * alpha) / g |
| Ball trajectory (conversion from degrees to radians): | X_g = vi^2^ * sin(pi * alpha / 90) / g |
| Distance between target and ball landing (error): | Error = zeros (size*X_g)) + (X_g < tgt_range(1)) * (tgt_range(1) – X_g) + (X_g > tgt_range(2)) * (X_g – tgt_range(2)) |
